# Role of apoptosis-related miRNAs in resveratrol-induced breast cancer cell death

**DOI:** 10.1038/cddis.2016.6

**Published:** 2016-02-18

**Authors:** R Venkatadri, T Muni, A K V Iyer, J S Yakisich, N Azad

**Affiliations:** 1 Department of Pharmaceutical Sciences, Hampton University, Hampton, VA, USA

## Abstract

Breast cancer is the most frequently diagnosed cancer in women, and one of the leading causes of cancer-related deaths worldwide. Recent evidences indicate that dietary agents such as resveratrol may inhibit cancer progression through modulation of microRNAs (miRNAs). We demonstrate that resveratrol regulates apoptotic and cell cycle machinery in breast cancer cells by modulating key tumor-suppressive miRNAs including miR-125b-5p, miR-200c-3p, miR-409-3p, miR-122-5p and miR-542-3p. Resveratrol-mediated miRNA modulation regulates key anti-apoptotic and cell cycle proteins including Bcl-2, X-linked inhibitor of apoptosis protein and CDKs, which are critical for its activity. Modulating miRNAs with mimics or inhibitors further validated a key role for miR-542-3p in MCF-7 and miR-122-5p in MDA-MB-231 breast cancer cell death in response to resveratrol. In conclusion, this study reveals novel miRNAs modulated by resveratrol that have a key role in breast cancer cell death.

Breast cancer is the second most common type of cancer in women, and the fifth most common cause of cancer-related deaths in the world. Nearly 200 000 women get diagnosed and about 40 000 die of breast cancer every year worldwide.^1^ Prolonged use of chemotherapeutic drugs against breast cancer mostly renders the drug ineffective because of development of resistance against the therapeutic agents. Identifying alternative treatments is crucial to reduce the mortality rate related to breast cancer.

Cell cycle arrest and apoptosis are considered important for therapeutics targeting cancer cells. It is often observed that cancer cells have altered cell cycle machinery. During the transition of a normal cell to cancerous state, cyclin-dependent kinases (CDKs) that govern coordinated initiation, progression and completion of cell cycle are overexpressed causing uncontrolled abnormal cell growth.^2^ Apoptosis or programmed cell death occurs naturally in all tissues to maintain tissue homeostasis and acts as a mechanism to eliminate unwanted cells. Cell division through the quiescence (G0) to the proliferative phases is controlled by the cell cycle. The DNA synthesis phase (S phase) and the mitosis (M phase) are separated by the G1 and G2 phases. Several drugs targeting the cell cycle have entered clinical trials and some of the well-known drugs currently used exhibit their effects by targeting the cell cycle. Cell cycle arrest is known to cause apoptosis and cell death in human malignancies.^3, 4^ Apoptosis occurs via two controlled pathways: the extrinsic or death receptor-mediated pathway, which activates caspase-8; and the intrinsic or mitochondria-mediated pathway, which activates caspase-9. These caspases known as initiator caspases activate downstream effector caspases (caspase-3, -6, and -7), which induce cleavage of several key cellular proteins to activate cell death. Cancer therapies like chemotherapy and many anticancer drugs primarily act by inducing apoptosis.

Natural plant-derived compounds, including resveratrol have been reported to induce apoptosis and cell cycle arrest in tumor cells.^5, 6, 7^ Resveratrol is a dietary agent found in a wide variety of plants like grapes, berries and peanuts and is known to have antioxidant and anti-inflammatory properties. It is emerging as a promising anticancer agent because of its chemopreventive and pro-apoptotic properties.^8, 9, 10, 11^ Resveratrol has been shown to have a crucial role in apoptosis induction in human breast cancer cells.^12, 13^ Moreover, studies show that several members of the mitogen-activated protein kinase signaling pathway are involved in this activation^14^ and the intrinsic mitochondrial pathway, via activation of caspase-9 along with other key mediators calcium and calpain, is the major pathway involved in resveratrol-induced apoptosis.^15^

MicroRNAs (miRNAs) are emerging as potential diagnostic, prognostic and therapeutic tools for breast cancer treatment.^16, 17^ MiRNAs are small non-coding single-stranded RNAs that negatively regulate gene expression by binding to mRNA and inhibiting translation. They control normal cell functions like cell cycle regulation, proliferation, differentiation and apoptosis. They have been implicated to have a critical role in the development and progression of various types of cancers including breast cancer. Owing to their significant and versatile roles, miRNAs are emerging as therapeutic tools for many cancers. Several miRNAs have been shown to be dysregulated in breast cancer tissues when compared with normal tissues.^18^ Modulation of tumor-suppressive miRNA by natural chemopreventive agents such as resveratrol has been shown to induce cell death via apoptosis in various cancer cells including prostate cancer cells.^19^ Interestingly, a link between resveratrol-induced apoptosis and miRNA modulation in relation to breast cancer has not been studied.

In this study, we investigated the anti-proliferative effects of miRNA modulation by resveratrol in breast cancer cells. We identified novel tumor-suppressive miRNAs differentially regulated by resveratrol in MCF-7 and MDA-MB-231 breast cancer cells that regulate apoptosis and cell cycle machinery. Furthermore, the rate-limiting miRNAs and their key target proteins that could serve as potential targets for tumor inhibition were identified. Elucidating the effect of resveratrol-mediated miRNA modulation during breast cancer cell death may aid in better understanding of the underlying mechanisms that have a critical role in breast cancer.

## Results

### Caspase activation and apoptosis induction by resveratrol in breast cancer cells

**Figure 1:**
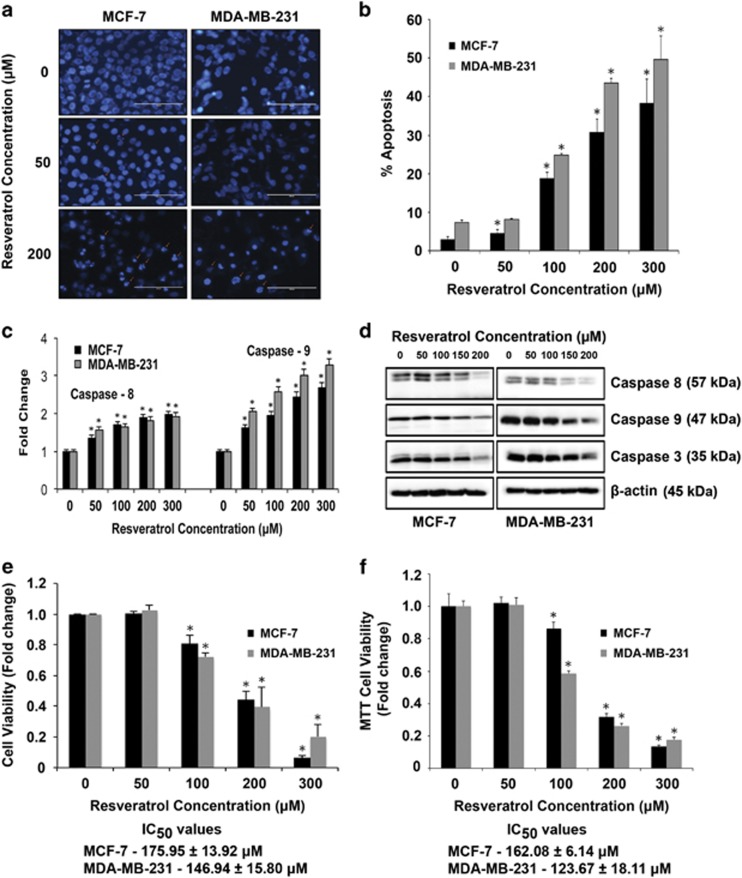
Resveratrol induces apoptosis in breast cancer cells. (**a**) Representative fluorescence micrographs of MCF-7 and MDA-MB-231 cells treated with resveratrol (0–200 *μ*M) for 24 h showing intensely condensed chromatin and/or fragmented nuclei (shown by arrows); scale bar, 100 *μ*M. (**b**) MCF-7 and MDA-MB-231 cells were treated with indicated concentrations of resveratrol for 24 h and analyzed for apoptosis by Hoechst 33342 assay. (**c**) Fluorometric assay of caspase activity in cells treated with resveratrol (0–300 *μ*M) for 12 h. Cell lysates (50 *μ*g of protein) were prepared and analyzed for caspase activity using the fluorometric substrates IETD-AMC and LEHD-AMC for caspase-8 and -9, respectively. Plots show relative fluorescence intensity over untreated control. (**d**) Cell lysates (30 *μ*g proteins) from resveratrol (0–200 *μ*M) treated MCF-7 and MDA-MB-231 cells were analyzed for caspase activation by western blotting. Representative data from three or more independent experiments are shown. (**e**) MCF-7 and MDA-MB-231 cells were treated with indicated concentrations of resveratrol for 24 h and cell viability was assessed by CellTiter-Glo Luminescent Cell Viability Assay. (**f**) MCF-7 and MDA-MB-231 cells were treated with indicated concentrations of resveratrol for 24 h and assessed by MTT Assay. Data represent mean values±S.E.M. of triplicate determinations from three or more independent experiments. *P<0.05 *versus* untreated control

We first characterized the apoptotic response to resveratrol treatment in MCF-7 and MDA-MB-231 human breast cancer cell lines. Cells were treated with various concentrations of resveratrol (0–300 *μ*M) and apoptosis was determined after 24 h by Hoechst assay. Figures 1a and b show that resveratrol treatment caused a dose-dependent increase in apoptosis over control level, as indicated by increased nuclear fluorescence and chromatin condensation of the treated cells. Furthermore, caspase activity assays using specific enzyme substrates for caspase-8 and -9 showed a dose-dependent increase in caspase-8 and -9 activities in response to resveratrol in both MCF-7 and MDA-MB-231 cells (Figure 1c). The effect was more pronounced in MDA-MB-231 cells. Caspase activation and apoptosis induction was further confirmed by western blotting for various caspases (Figure 1d). Both cell lines were similarly treated with various concentrations of resveratrol for 24 h and analyzed for cell viability (Figures 1e and f). Resveratrol treatment significantly decreased cell viability in a dose-dependent manner in both MCF-7 and MDA-MB-231 cells. The IC_50_ values were 162.08±6.14 and 123.67±18.11 *μ*M as determined by the MTT assay.

### Resveratrol regulates apoptosis-related miRNAs in breast cancer cells

MCF-7 and MDA-MB-231 cells were treated with resveratrol and 12 h post-treatment real-time PCR amplification of isolated RNA using human apoptosis miRNA array, which analyzes a panel of 84 miRNAs known to be associated with cellular apoptosis was performed. Data analysis using the Qiagen (Valencia, CA, USA) online data analysis software revealed many miRNAs to be regulated as a result of apoptotic effect of resveratrol treatment *versus* control samples (Supplementary Table 1). Interestingly, most miRNAs from the panel of tested miRNAs were found to be downregulated in both cell lines. Table 1 shows miRNAs regulated by more than twofold in response to resveratrol treatment and miR-409-3p (1.6-fold change), which was the only one upregulated in MCF-7 cells. Thirty-five miRNAs were found to be downregulated in MCF-7 cells by more than twofold, among which miR-542-3p (over eightfold) and miR-125b-5p (over fivefold) showed the largest changes. In MDA-MB-231 cells, of the upregulated miRNAs, miR-122-5p showed a 37.6-fold change. Twenty-five miRNAs were found to be downregulated by >2-fold, among which miR-542-3p (over 11-fold) and miR-200c-3p (over 8-fold) showed the largest changes.

### Resveratrol-mediated miRNA regulate key apoptosis and cell cycle proteins

**Figure 2:**
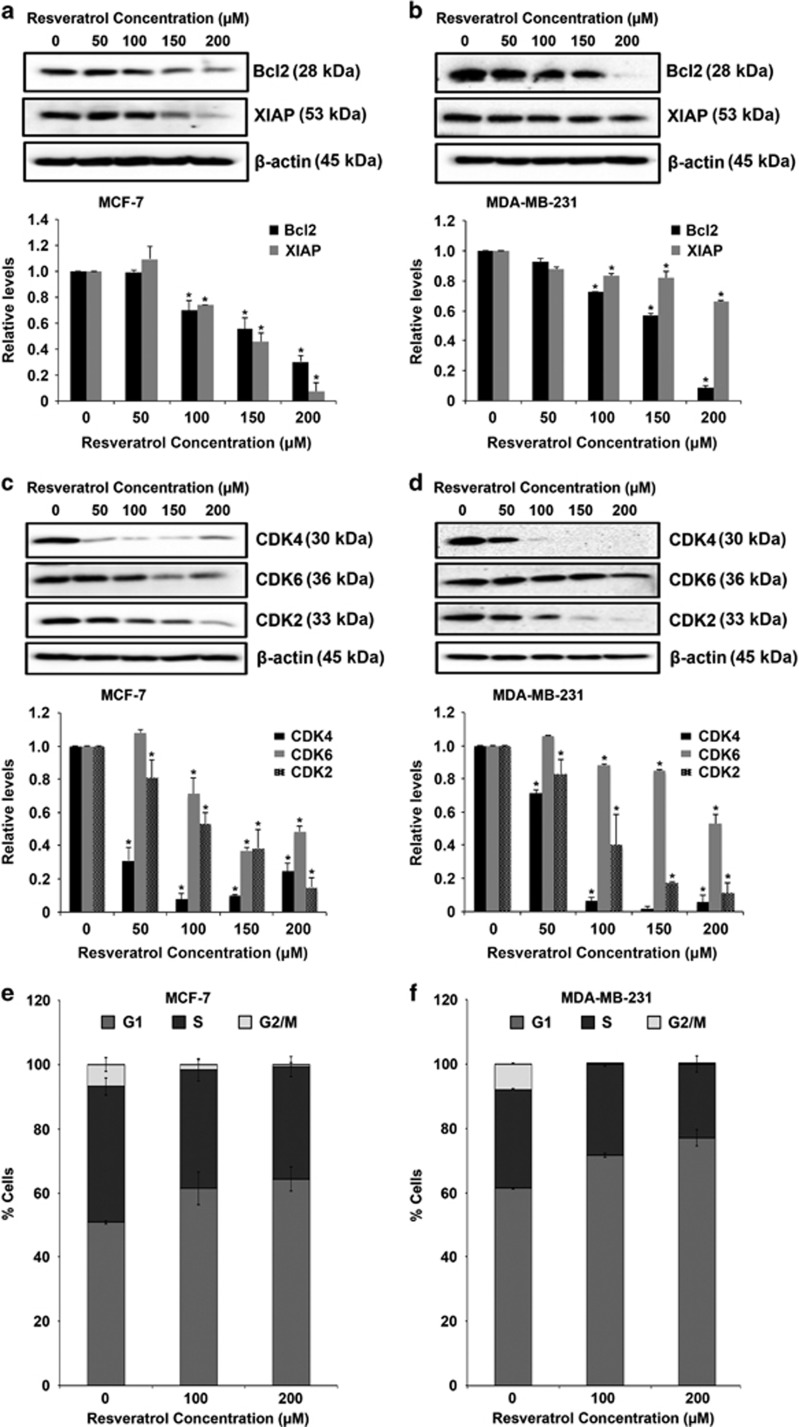
Resveratrol-mediated regulation of key apoptosis and cell cycle proteins. (**a**) MCF-7 and (**b**) MDA-MB-231 cells were treated with resveratrol (0–200 *μ*M) for 24 h. Cell lysates were collected and analyzed for Bcl-2 and XIAP protein expression. (**c**) MCF-7 and (**d**) MDA-MB-231 cells were treated with resveratrol (0–200 *μ*M) for 24 h and cell lysates were analyzed for CDK4, CDK6 and CDK2. All blots were reprobed with *β*-actin antibody to confirm equal loading of the samples. The immunoblot signals were quantified by densitometry. Values are mean±S.E.M. (*n*=3). *P<0.05 *versus* untreated control. (**e**) MCF-7 and (**f**) MDA-MD-231 cells treated with resveratrol were analyzed for cell cycle distribution using NovoCyte flow cytometer and ModFit 4.0.5 LT. Data represent mean values±S.E.M. from three independent experiments

For both MCF-7 and MDA-MB-231 cell lines, miRNAs that were most regulated (maximum fold change) by resveratrol treatment were selected for further analysis. Bioinformatics analysis with the aid of miRNA databases revealed as well as predicted experimentally validated targets of the identified miRNA (Table 2). Interestingly cyclins, caspases, Bcl-2 and many Bcl-2-like genes were targets for all miRNAs in both cell lines. The results of bioinformatics analysis were validated by western blot analysis. MCF-7 and MDA-MB-231 cells were treated with various concentrations of resveratrol and protein expression was analyzed. Resveratrol treatment caused a dose-dependent downregulation of anti-apoptotic proteins including Bcl-2 and X-linked inhibitor of apoptosis protein (XIAP) and cell cycle proteins including CDK2, CDK4 and CDK6 (Figures 2a, b, c and d). Furthermore, cell cycle analysis was performed to determine the distribution of different phases of cells using flow cytometry. Resveratrol treatment induced a dose-dependent G1-arrest in both MCF-7 and MDA-MB-231 cells. Comparatively, there was lower percentage of cells in the S phase and significantly lower percentage in the G2/M phase for both MCF-7 and MDA-MB-231 cells (Figures 2e and f).

### Modulation of key miRNAs regulates resveratrol-mediated apoptosis

**Figure 3:**
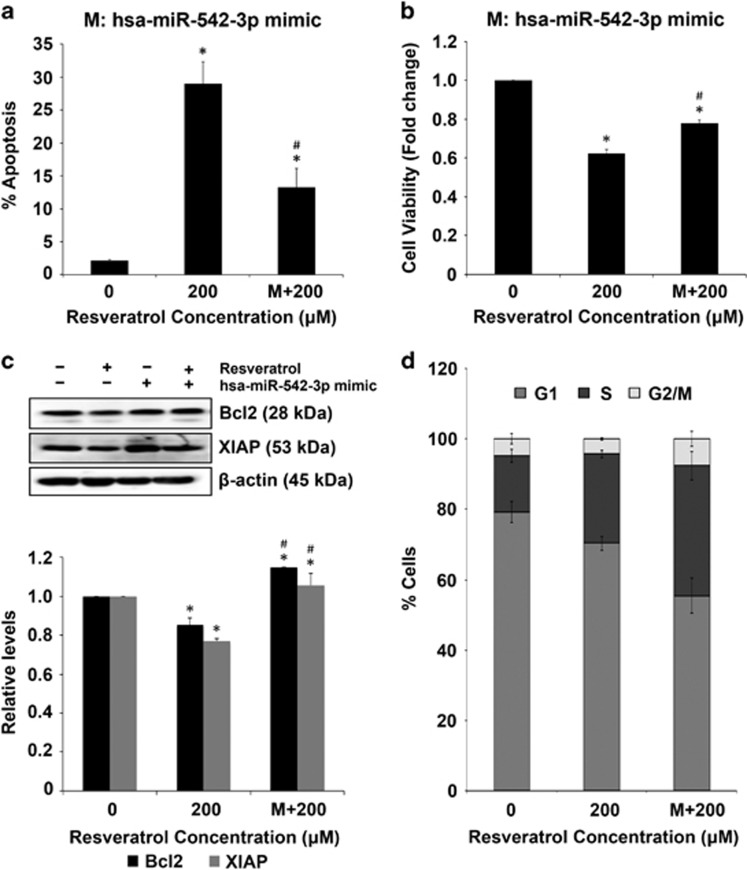
Effect of miR-542-3p modulation in MCF-7 cells. (**a**) MCF-7 cells were transfected with miR-542-3p mimic (100 nM) for 40 h and then treated with resveratrol for 24 h. Apoptosis induction was assessed by Hoechst 33342 staining. (**b**) MCF-7 cells were transfected with miR-542-3p mimic (100 nM) for 40 h and then treated with resveratrol for 24 h. Cell lysates were analyzed for cell viability using CellTiter-Glo Luminescent assay. Plots are mean±S.E.M. (*n*=3). *P<0.05 *versus* untreated control. ^#^*P*<0.05 for resveratrol-treated cells *versus* resveratrol-treated cells transfected with the miRNA mimic. (**c**) MCF-7 cells were transfected with miR-542-3p mimic (100 nM) for 40 h and then treated with resveratrol for 24 h. Cell lysates were analyzed for Bcl-2 and XIAP expression levels by western blotting. Blots were reprobed with *β*-actin antibody to confirm equal loading of the samples. The immunoblot signals were quantified by densitometry. Values are mean±S.E.M. (*n*=3). *P<0.05 *versus* untreated control. ^#^*P*<0.05 for resveratrol-treated cells *versus* resveratrol-treated cells transfected with the miRNA mimic. (**d**) Cell cycle analysis of MCF-7 cells transfected with miR-542-3p mimic (100 nM) for 40 h and then treated with resveratrol for 24 h. Plots are mean±S.E.M. (*n*=4)

**Figure 4:**
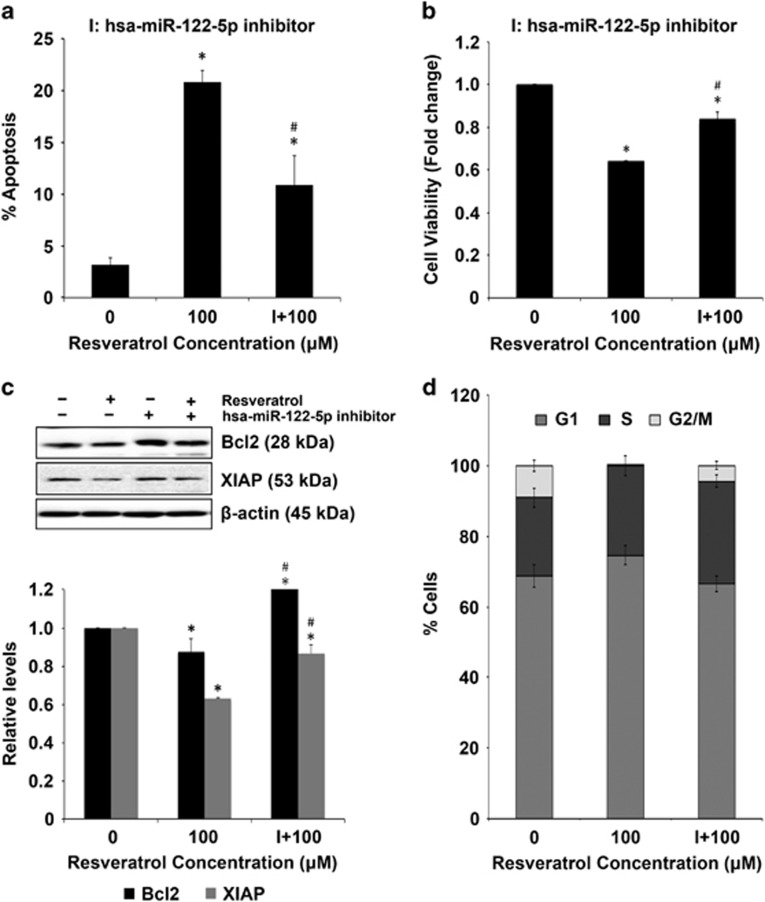
Effect of miR-122-5p modulation in MDA-MB-231 cells. (**a**) MDA-MB-231 cells were transfected with miR-122-5p inhibitor (100 nM) for 40 h and then treated with resveratrol for 24 h. Apoptosis induction was assessed by Hoechst 33342 staining. (**b**) MDA-MB-231 cells were transfected with miR-122-5p inhibitor (100 nM) for 40 h and then treated with resveratrol for 24 h. Cell lysates were analyzed for cell viability using CellTiter-Glo Luminescent assay. Plots are mean±S.E.M. (*n*=3). *P<0.05 *versus* untreated control. ^#^*P*<0.05 for resveratrol-treated cells *versus* resveratrol-treated cells transfected with the miRNA inhibitor. (**c**) MDA-MB-231 cells were transfected with miR-122-5p inhibitor (100 nM) for 40 h and then treated with resveratrol for 24 h. Cell lysates were analyzed for Bcl-2 and XIAP expression levels by western blotting. Blots were reprobed with *β*-actin antibody to confirm equal loading of the samples. The immunoblot signals were quantified by densitometry. Values are mean±S.E.M. (*n*=3). *P<0.05 *versus* non-treated control. ^#^*P*<0.05 for resveratrol-treated cells *versus* resveratrol-treated cells transfected with the miRNA inhibitor. (**d**) Cell cycle analysis of MDA-MB-231 cells transfected with miR-122-5p inhibitor (100 nM) for 40 h and then treated with resveratrol for 24 h. Plots are mean±S.E.M. (*n*=4)

MCF-7 cells were transfected with miR-542-3p mimic and treated with resveratrol for 24 h, followed by assessment of apoptosis and cell viability. Transfection with miR-542-3p mimic significantly reversed the effect of resveratrol on apoptosis and viability of MCF-7 cells as well as Bcl-2 and XIAP protein levels (Figures 3a, b and c). Interestingly, the percentage of MCF-7 cells transfected with miR-542-3p mimic treated with resveratrol was lower in the G1 phase and higher in S and G2/M phases as compared with non-transfected MCF-7 cells treated with resveratrol (Figure 3d). MDA-MB-231 cells were transfected with miR-122-5p inhibitor and treated with resveratrol for 24 h. Transfection with miR-122-5p inhibitor reversed the effect of resveratrol on apoptosis, cell viability and Bcl-2 and XIAP proteins (Figures 4a, b and c). Similar to MCF-7 cells, MDA-MB-231 cells transfected with miR-122-5p inhibitor treated with resveratrol showed lesser cells in G1 phase and more cells in S and G2/M phases as compared with non-transfected MDA-MB-231 cells treated with resveratrol (Figure 4d).

### Effect of resveratrol on breast cancer miRNAs

**Figure 5:**
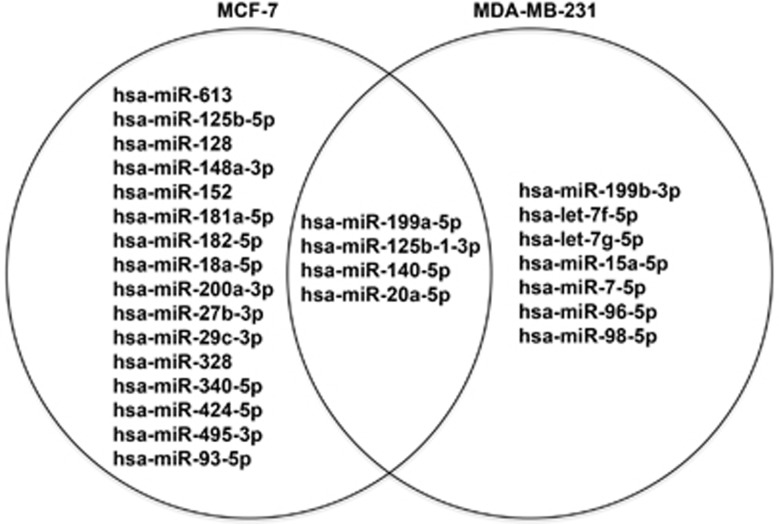
Venn diagram showing breast cancer miRNA microarray data for MCF-7 and MDA-MB-231 cells treated with 200 *μ*M resveratrol for 12 h as compared with untreated control cells that show at least a twofold change in miRNA that are differentially regulated

**Figure 6:**
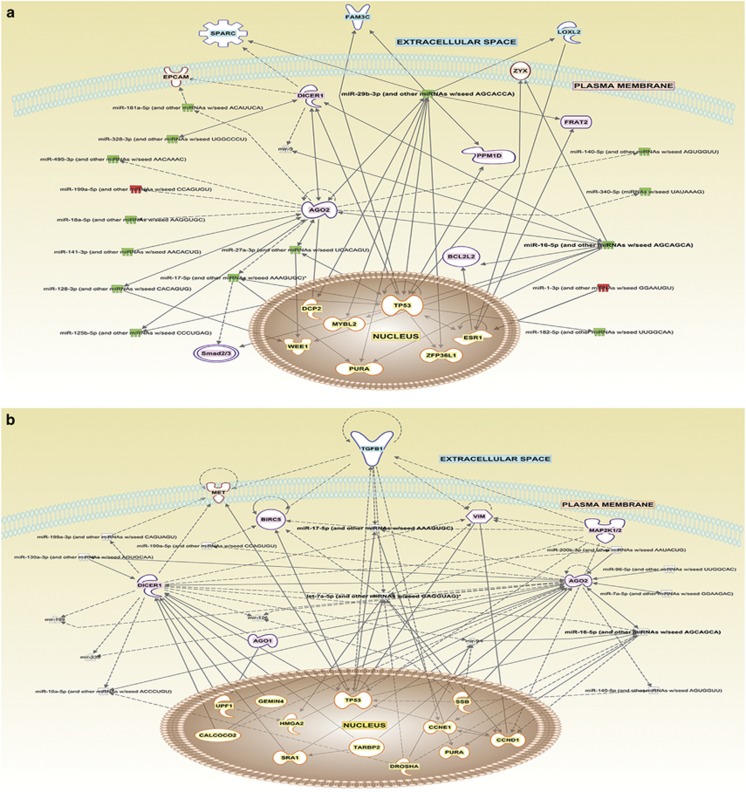
Path design analysis of breast cancer-related miRNAs. Network maps of important biomarkers predicted to have a key role in breast cancer in response to resveratrol treatment were generated using IPA. Potential protein and miRNA interactions were visualized using the Path Design Suite for both (**a**) MCF-7 and (**b**) MDA-MB-231 cells. Dotted lines connect genes that are known to be affected indirectly and solid lines connect genes that are known to be directly affected by resveratrol treatment

Modulation of breast cancer-related miRNAs by resveratrol in MCF-7 and MDA-MB-231 cells was investigated by quantitative real-time PCR using a human breast cancer array, which analyzes a panel of 84 miRNAs known to be associated with breast cancer (Supplementary Table 2). The data were analyzed using the miScript miRNA PCR Array Data Analysis portal (Qiagen), and a clustergram of the array data for each cell line was generated (Supplementary Figure 1). Breast cancer miRNAs regulated by more than twofolds in response to resveratrol treatment is shown in Figure 5 and Supplementary Table 3. Two miRNAs were upregulated in both MCF-7 and MDA-MB-231 cells by more than twofolds, of which miR-199a-5p was common to both. A total of 18 miRNAs in MCF-7 and 9 miRNAs in MDA-MB-231 cells were downregulated by more than twofolds with resveratrol treatment. Notable miRNAs that were common in both cell lines were miR-199a-5p, miR-125b-1-3p, miR-140-5p and miR-20a-5p. The expression profiles of miRNAs identified for each cell line were subjected to ingenuity pathways analysis (IPA), and a network map of potential proteins and pathways were generated using path design within the IPA core analysis tool for both MCF-7 and MDA-MB-231 cells (Figures 6a and b). IPA shows that several key apoptotic proteins already identified in this study form the central nodes of the network pathways predicted for both MCF-7 and MDA-MB-231 cells.

## Discussion

Naturally occurring chemopreventive agents have gained significant interest as reports of their effect on modulating miRNAs to inhibit cancer growth and metastasis are becoming evident.^20, 21, 22, 23^ Resveratrol is known to induce apoptosis in breast cancer cells.^24, 25, 26, 27^ We validated the effect of resveratrol on cell viability and apoptosis in MCF-7 and MDA-MB-231 cells (Figure 1). Although resveratrol-induced activation of both caspase-8 and -9, we observed that the mitochondrial caspase-9 pathway was the major apoptotic pathway involved in both MCF-7 and MDA-MB-231 cells (Figures 1c and d). Caspases are known to control the cell regulatory function that initiates the process of apoptosis and are an important feature of therapeutics that target cancer.^28^ Our observation also confirms previous reports on the intrinsic mitochondrial pathway being the major pathway involved in resveratrol-induced apoptosis, via activation of caspase-9.^15^ Resveratrol has also been reported to activate the extrinsic pathway of cell death, including FAS-dependent apoptosis in HL-60 pro-myelocytic leukemia cells^29, 30^ and TRAIL-induced apoptosis in prostate cancer cells.^31^ Furthermore, resveratrol exerted its pro-apoptotic effects by downregulating expression of key anti-apoptotic proteins belonging to the inhibitor of apoptosis (IAPs) family of proteins Bcl-2 and XIAP (Figures 2a and b) and also decreased expression of CDKs specific for G-phase arrest (Figures 2c and d). Cell cycle analysis by flow cytometry clearly showed a dose-dependent G1-arrest for both MCF-7 and MDA-MB-231 cells induced by resveratrol (Figures 2e and f). Cell cycle regulation upon treatment with resveratrol seems to be cell-type specific – whereas resveratrol induces G0/G1-arrest in prostate cancer cells and leukemic cells,^32, 33, 34, 35^ Pozo-Guisado *et al.*^36^ reported no significant changes in cell cycle distribution of MDA-MB-231 cells treated with resveratrol treatment. Such differences may also be attributed to variations in experimental conditions used in these studies. Several studies have focused on utilizing small molecule inhibitors and molecules to specifically target IAPs and CDKs and this has been proven as a successful strategy for cancer treatment.^37, 38, 39^

We tested the effect of resveratrol on apoptosis-related miRNAs using human apoptosis miRNA array. MiRNAs are reported to have a pivotal role in breast cancer progression, functioning either as oncogenes or tumor-suppressor genes. MiRNAs have been shown to be dysregulated in breast cancer tissues as compared with normal tissues,^40^ and therefore could be important targets in cancer treatment. Several miRNAs with altered expression patterns are identified in various types of cancers.^41, 42^ MiRNAs thus serve as therapeutic targets for cancer treatment because of their effect on multiple target genes and proteins. We report several miRNAs that were differentially regulated by resveratrol in MCF-7 and MDA-MB-231 cells (Table 1). Online databases such as TargetScan, miRBase and miRanda were used to identify major protein targets of the shortlisted miRNAs (Table 2).^43, 44, 45, 46^ Two miRNAs in MCF-7 (miR-125b-5p and miR-542-3p) and two miRNAs in MDA-MB-231 (miR-200c-3p and miR-542-3p) were downregulated by over fivefolds. MiR-409-3p and miR-122-5p were the only upregulated miRNAs in MCF-7 and MDA-MB-231 cells, respectively. MiR-542-3p was downregulated in both the cell lines. Interestingly, all the shortlisted miRNAs were predicted to primarily target caspases, anti-apoptotic protein Bcl-2 and cell cycle-related cyclins (Table 2).

To investigate the role of miRNA modulation in resveratrol-induced apoptosis of breast cancer cells, one miRNA that was the most affected by resveratrol treatment for each cell line was selected for further validation. Based on their maximum fold changes, miR-542-3p for MCF-7 and miR-122-5p for MDA-MB-231 cells were selected. Chemically synthesized miRNA mimic and inhibitors were used to validate miRNAs and study their effects on tumor inhibition.^47, 48^ MCF-7 cells transfected with miR-542-3p mimic reversed the effect of resveratrol on apoptosis and the anti-apoptotic proteins Bcl-2 and XIAP (Figure 3). Recent studies have shown that downregulation of miR-542-3p is associated with cancer invasiveness and resistance to therapy.^49, 50, 51^ Yoon *et al.*^52^ reported that the observed tumor-suppressive function of this miRNA is by targeting the anti-apoptotic protein survivin. In our study, we found that resveratrol downregulates miR-542-3p, which is imperative for the observed tumor inhibitory effects. Individual miRNA can be controlled by other miRNA as part of miRNA clusters, which varies in each system. This observation is particularly interesting because it highlights complex interactions among miRNAs that are yet to be understood. MDA-MB-231 cells transfected with miR-122-5p inhibitor showed similar resistance to resveratrol-induced apoptosis and reversed its effect on anti-apoptotic proteins (Figure 4). MiR-122-5p has been reported to regulate tumorigenesis in hepatocellular carcinoma^53^ and to have tumor-suppressive function in breast cancer.^54^ Cell cycle analysis further revealed that the observed effects of resveratrol are through the identified miRNAs as there were lower percentage of MCF-7 and MDA-MB-231 cells transfected with miRNA modulators in the G1 phase in response to resveratrol treatment. As compared with data in response to direct resveratrol treatment presented in Figure 2, the experimental and culture conditions are different in Figures 3 and 4, where cells were transfected with miRNA mimics or inhibitors before treating with resveratrol. Therefore, the effect observed on apoptosis, viability, cell cycle arrest, Bcl-2 and XIAP proteins is less pronounced in cells, which were transfected before resveratrol treatment (Figures 3 and 4) as compared with direct treatment to resveratrol (Figure 2).

We also performed miRNA expression profiling using Breast Cancer arrays to identify key miRNAs known to be associated with breast cancer progression in response to resveratrol treatment (Figure 5 and Supplementary Table 3). Several miRNAs were modulated in MCF-7 and MDA-MB-231 cells in response to resveratrol treatment, of which four miRNAs associated with breast cancers including miR-199a-5p, miR-125b-1-3p, miR-140-5p and miR-20a-5p were common in both cell lines. These miRNAs have been widely reported to have a role in cancer initiation, progression and inhibition. MiR-199a-5p has been reported to suppress liver cancer by inhibiting glucose metabolism, regulate autophagic response in breast cancer and function as an oncogene in the development of gastric cancer.^55, 56, 57^ The miR-125b, miR-140 and miR-17/20 clusters are also reported to inhibit breast cancer progression.^58, 59, 60^ Validation of these miRNAs warrants further investigation and is beyond the scope of this study, which focuses on cell death and cell cycle pathways. Interestingly, miR-125b-1-3p is the only miRNA observed to be downregulated by resveratrol in both the apoptotic and breast cancer arrays. This provides extensive scope for further investigations as downregulation of miR-125b has been observed in head and neck cancer development and breast cancer progression.^61, 62^

Overall, our findings demonstrate that resveratrol-induced apoptosis in MCF-7 and MDA-MB-231 cells and showed a dose-dependent effect on cell viability. Resveratrol mediated its apoptotic effect via caspase activation and downregulation of anti-apoptotic proteins including XIAP and Bcl-2. Interestingly, resveratrol-modulated apoptotic miRNAs had a key role in mediating its effect on breast cancer cell death. Overall, our study reveals a novel role for resveratrol in inhibiting breast cancer progression by modulating tumor-suppressive miRNAs and thereby affecting cell cycle and apoptosis. We report novel miRNAs including miR-542-3p and miR-122-5p to have a key role in resveratrol-mediated effects on MCF-7 and MDA-MB-231 breast cancer cells, respectively. Evaluating the potential of resveratrol to modulate epigenetic events associated with breast cancer could facilitate in the discovery and development of novel therapeutic strategies against this disease.

## Materials and Methods

### Chemicals and reagents

Resveratrol was obtained from Sigma-Aldrich (St. Louis, MO, USA). A 100 mM stock was prepared in ethanol and stored at 4°C until further use. All antibodies including caspase-8, caspase-9, caspase-3, Bcl-2, XIAP, HRP-conjugated anti-rabbit IgG and anti-mouse IgG antibodies was purchased from Cell Signaling Technology (Danvers, MA, USA). *β*-Actin antibody, Hoechst 33342 and propidium iodide was purchased from Sigma-Aldrich.

### Cell culture

Human breast adenocarcinoma cells MCF-7 and MDA-MB-231 were obtained from American Type Culture Collection (Manassas, VA, USA). Cells were cultured in Dulbecco's Modified Eagle medium (Thermo Scientific, Waltham, MA, USA) supplemented with 10% FBS, 2 mM l-glutamine, 100 U/ml penicillin and 100 mg/ml streptomycin in a 5% CO_2_ environment at 37 °C.

### Apoptosis assay

Apoptosis was determined by Hoechst 33342 DNA fragmentation assay. Briefly, cells were incubated with 10 *μ*g/ml Hoechst 33342 nuclear stain (Life Technologies, Carlsbad, CA, USA) for 30 min at 37 °C and percentage of cells having intensely condensed chromatin and/or fragmented nuclei by fluorescence microscopy (EVOS All-in-one digital inverted fluorescence microscope, Thermo Fisher Scientific, Waltham, MA, USA) were scored. From random fields, nuclei were analyzed for each sample. The apoptotic index was calculated as apoptotic nuclei/total nuclei x 100 (%) using ImageJ software (Java image processing, NIH, Bethesda, MD, USA).

### Caspase assay

Caspase activity was determined by fluorometric assay using the enzyme substrate IETD-AMC for caspase-8 and LEHD-AMC for caspase-9, which are specifically cleaved by the respective enzymes at the Asp residue to release the fluorescent group, AMC. Cell extracts containing 50 *μ*g of protein were incubated with 100 mM HEPES containing 10% sucrose, 10 mM dithiothreitol, 0.1% 3-[(3-cholamidiopropyl)-dimethylammonio]-1-propane sulfonate and 50 mM caspase substrate in a total reaction volume of 0.25 ml. The reaction mixture was incubated for 60 min at 37 °C, and quantified fluorometrically at the excitation and emission wavelengths of 380 and 460 nm, respectively (Synergy H1 Hybrid Reader, BioTek, Winooski, VT, USA).

### CellTiter-Glo luminescent cell viability assay

Breast cancer cells (MCF-7 and MDA-MB-231) treated with resveratrol were assessed for cell viability using CellTiter-Glo Luminescent cell viability assay (Promega, Madison, WI, USA) following the manufacturer's instructions. Briefly, 1 × 10^4^ cells per well were seeded on a 96-well plate and the cells were incubated overnight. Luminescence was measured after cells were treated for 24 h and lysed with 50 *μ*l CellTiter-Glo reagent for 10–15 min (Synergy H1 Hybrid Reader, BioTek).

### MTT assay

Breast cancer cells (MCF-7 and MDA-MB-231) were seeded in 96-well plates and treated with indicated concentrations of resveratrol for 24 h. Post-treatment, 20 *μ*l per well of MTT (5 mg/ml in PBS) was added and the plates were incubated at 37 ºC. DMSO (200 *μ*l per well) was added and the dark blue formazan product was quantified by measuring absorbance at 570 nm (with a 690 nm reference filter; Synergy H1 Hybrid Reader, BioTek).

#### MiRNA expression analysis and qRT-PCR

Isolation of RNA: total RNA was isolated using the miRNeasy mini kit (Qiagen) according to the manufacturer's instructions. The quality of RNA was assessed on a native agarose gel by analyzing the 18S and 28S ribosomal RNA bands and by measuring purity at 260 and 280 (data not shown).
miRNA amplification: cDNA synthesis was performed using the miScript II RT kit (Qiagen) as per the manufacturer's instructions. Real-time PCR amplification of the cDNA was performed using miScript SYBR Green PCR kit (Qiagen) for known apoptosis-related miRNA using Human Apoptosis miRNA PCR arrays (Qiagen). cDNA conversion and real-time PCR were performed as per the manufacturer's instructions.
PCR array data analysis: data sets for each sample were collected at the same threshold and baseline levels to maintain consistency between samples for further analysis. The PCR array data were analyzed using the web-based data analysis software offered by Qiagen (http://pcrdataanalysis.sabiosciences.com/mirna/arrayanalysis.php). A twofold change cut-off was selected to identify miRNA that were significantly altered because of resveratrol treatment. The accession number and sequence of the identified miRNA were obtained using the miRBase (www.mirbase.org) database. miRWalk (http://www.umm.uni-heidelberg.de/apps/zmf/mirwalk/index.html) was used to obtain a list of predicted and validated miRNA targets from apoptosis-related pathways using the following pathway (database): Apoptosis (KEGG), Caspase (Biocarta), Death (Biocarta) and Mitochondria (Biocarta).

### Transient transfection

MCF-7 and MDA-MB-231 cells were seeded in six-well culture plates. The cells were transfected in complete medium with miRNA mimic, miRNA inhibitor or control (AllStars Hs Cell Death Control siRNA) (Qiagen) using Lipofectamine 3000 (Invitrogen, Carlsbad, CA, USA) transfecting agent according to the manufacturer's protocol. Briefly, lipofectamine (15 *μ*l) was added to miRNA mimic or miRNA inhibitor (100 nM) diluted with 50 *μ*l serum-free medium. This complex was incubated for 15 min and then added to the cells. After 40 h, the medium was discarded and the cells were washed with PBS and treated with resveratrol in serum-free medium.

### Flow cytometry

MCF-7 and MDA-MB-231 cells were seeded in six-well culture plates and were treated in complete medium for cell cycle analysis. After 24 h, cells were trypsinized and suspended in 70% ethanol overnight. The cells were stained with PI and data acquisition was performed in a NovoCyte flow cytometer (ACEA Biosciences, Inc., San Diego, CA, USA) using NovoExpress 1.0.2 software. For transfection experiments, cells were transfected in complete medium for 40 h and then treated in serum-free medium for 24 h.

### Gating strategy

Acquired samples were initially screened with SSC-H *versus* FSC-H density plot in linear scale and the gates were set to exclude debris. Another density plot was generated with the cells in the set gates and analyzed in PE-H *versus* PE-A for doublet discrimination. Singlet cells were gated, exported as FCS files and analyzed using ModFit 4.0.5 LT cell cycle analysis software (Verity Software House, Topsham, ME, USA).

### Western blotting

After specific treatments, cell lysates were resolved on a 10% sodium dodecyl sulfate-polyacrylamide gel electrophoresis and transferred onto a nitrocellulose membrane. The protein concentration was determined using a bicinchoninic acid protein assay kit (Pierce Biotechnology, Rockford, IL, USA), and equal amount of protein was loaded per sample. The membrane was blocked with TBS-T (0.1% Tween-20 in TBS) containing 5% dry milk, and incubated with primary antibody overnight at 4^o^C. After three washes with TBS-T, the membrane was incubated with HRP-conjugated secondary antibody for 1 h and then washed with TBS-T. The immune complexes were detected by chemiluminescence (Supersignal West Femto; Pierce Biotechnology) using MyECL Imager (Thermo Scientific), and quantified by using ImageJ (NIH, Image analysis using Java) digitizing software. Mean densitometry data from independent experiments were normalized to the control.

### Path design analysis

The IPA platform was used to discern global interaction networks associated with miRNA that were differentially regulated in MCF-7 and MDA-MB-231 cells. The miRNA data were uploaded into the software, and Network analysis was performed to identify important regulatory proteins associated with regulation of the miRNA. These protein pathways and miRNA were mapped out using the Path Design tool available to aid in visualization of interactions across various cellular compartments.

### Statistical analysis

Representative data from three or more independent experiments are shown as mean value±S.E.M. Statistical analysis was performed with two-way analysis of variance to identify differences between groups using GraphPad Prism Software (San Diego, CA, USA) and *P* values <0.05 considered significant.

## Figures and Tables

**Table 1:** Apoptosis miRNA microarray data for MCF-7 and MDA-MB-231 cells treated with 200 *μ*M resveratrol for 12 h as compared with untreated control cells showing at least a twofold change

ID	Function	Fold regulation
*MCF-7*		
hsa-miR-409-3p	Targets both pro- and anti-apoptotic genes	1.6057
hsa-let-7g-5p	Pro-apoptotic; targets anti-apoptotic genes	−2.4911
hsa-miR-101-3p	Pro-apoptotic; targets anti-apoptotic genes	−2.8615
hsa-miR-125b-5p	Targets both pro- and anti-apoptotic genes	−5.7231
hsa-miR-128	Pro-apoptotic	−2.8615
hsa-miR-143-3p	Pro-apoptotic	−3.0669
hsa-miR-17-5p	Targets both pro- and anti-apoptotic genes	−2.4911
hsa-miR-181a-5p	Anti-apoptotic, targets both pro- and anti-apoptotic genes	−2.3243
hsa-miR-181c-5p	Targets both pro- and anti-apoptotic genes	−3.523
hsa-miR-181d	Targets both pro- and anti-apoptotic genes	−2.6699
hsa-miR-183-5p	Anti-apoptotic	−2.1686
hsa-miR-186-3p	Anti-apoptotic	−2.1686
hsa-miR-192-5p	Targets both pro- and anti-apoptotic genes	−2.8615
hsa-miR-194-5p	Targets anti-apoptotic genes	−3.7758
hsa-miR-195-5p	Pro-apoptotic; targets anti-apoptotic genes	−3.2871
hsa-miR-210	Anti-apoptotic	−2.8615
hsa-miR-212-3p	Pro-apoptotic	−3.7758
hsa-miR-214-3p	Anti-apoptotic	−4.0468
hsa-miR-23a-3p	Either anti- or pro-apoptotic	−2.8615
hsa-miR-26b-5p	Pro-apoptotic	−3.0669
hsa-miR-29a-3p	Pro-apoptotic, targets both pro- and anti-apoptotic genes	−2.0234
hsa-miR-29b-3p	Either anti- or pro-apoptotic, targets both pro- and anti-apoptotic genes	−2.1686
hsa-miR-29c-3p	Pro-apoptotic, targets both pro- and anti-apoptotic genes	−3.0669
hsa-miR-30b-5p	Anti-apoptotic, targets both pro- and anti-apoptotic genes	−3.0669
hsa-miR-30c-5p	Pro-apoptotic	−2.0234
hsa-miR-31-5p	Pro-apoptotic	−3.523
hsa-miR-34a-5p	Pro-apoptotic, targets both pro- and anti-apoptotic genes	−2.4911
hsa-miR-34c-5p	Either anti- or pro-apoptotic	−3.0669
hsa-miR-378a-3p	Anti-apoptotic	−2.1686
hsa-miR-451a	Targets both pro- and anti-apoptotic genes	−3.7758
hsa-miR-497-5p	Pro-apoptotic, targets both pro- and anti-apoptotic genes	−2.1686
hsa-miR-512-5p	Pro-apoptotic; targets anti-apoptotic genes	−4.6486
hsa-miR-542-3p	Targets anti-apoptotic genes	−8.0937
hsa-miR-7-5p	Targets anti-apoptotic genes	−3.7758
hsa-miR-9-5p	Targets anti-apoptotic genes	−3.2871
hsa-miR-98-5p	Anti-apoptotic	−2.6699
		
*MDA-MB-231*		
hsa-miR-122-5p	Targets anti-apoptotic genes	37.6175
hsa-let-7a-5p	Either anti- or pro-apoptotic; targets both pro- and anti-apoptotic genes	−4.4898
hsa-miR-101-3p	Pro-apoptotic; targets anti-apoptotic genes	−2.9622
hsa-miR-106b-5p	Anti-apoptotic	−2.7638
hsa-miR-134	Targets pro-apoptotic genes	−5.5277
hsa-miR-141-3p	Anti-apoptotic	−3.1748
hsa-miR-143-3p	Pro-apoptotic	−3.1748
hsa-miR-146a-5p	Targets both pro- and anti-apoptotic genes	−3.4027
hsa-miR-15a-5p	Targets both pro- and anti-apoptotic genes	−2.5787
hsa-miR-16-5p	Pro-apoptotic, targets both pro- and anti-apoptotic genes	−2.2449
hsa-miR-181b-5p	Pro-apoptotic, targets both pro- and anti-apoptotic genes	−3.4027
hsa-miR-181d	Targets both pro- and anti-apoptotic genes	−2.2449
hsa-miR-183-5p	Anti-apoptotic	−2.5787
hsa-miR-186-3p	Anti-apoptotic	−3.9086
hsa-miR-192-5p	Targets both pro- and anti-apoptotic genes	−2.4061
hsa-miR-200c-3p	Pro-apoptotic	−8.3784
hsa-miR-212-3p	Pro-apoptotic	−2.4061
hsa-miR-26b-5p	Pro-apoptotic	−2.2449
hsa-miR-30b-5p	Anti-apoptotic, targets both pro- and anti-apoptotic genes	−3.9086
hsa-miR-32-5p	Targets pro-apoptotic genes	−4.8121
hsa-miR-34a-5p	Pro-apoptotic, targets both pro- and anti-apoptotic genes	−2.7638
hsa-miR-497-5p	Pro-apoptotic, targets both pro- and anti-apoptotic genes	−2.0946
hsa-miR-512-5p	Pro-apoptotic; targets anti-apoptotic genes	−2.9622
hsa-miR-542-3p	Targets anti-apoptotic genes	−11.0553
hsa-miR-7-5p	Targets anti-apoptotic genes	−4.1892
hsa-miR-98-5p	Anti-apoptotic	−2.7638

**Table 2:** Shortlisted resveratrol-induced miRNAs in MCF-7 and MDA-MB-231 breast cancer cells and their potential apoptotic targets

miRNA (SANGER ID)	Prediction format	p53	BCL2	BAX	FAS	Caspases	BRCA	Cyclins	Other
*MCF-7*									
hsa-miR-409-3p MIMAT0001639	Binding to 3' UTR		BCL2L15						
	Multiple prediction (DIANAmT, miRanda, miRDB, miRWalk, Targetscan)		BCL2, BCL2L2, BCL2L11, BCL2L13, BCL2L15			CASP2, CASP3, CASP4, CASP8, CASP10		Many cyclins and CDKs	
hsa-miR-542-3p MIMAT0003389	Binding to 3' UTR							CDKN1A	BIRC5
	Multiple prediction (DIANAmT, miRanda, miRDB, miRWalk, Targetscan)		BCL2, BCL2L15			CASP2, CASP6, CASP10		Many cyclins and CDKs	
hsa-miR-125b-5p MIMAT0000423	Binding to 3' UTR		BCL2L13, BCL2L14			CASP2		CCNJ	
	Multiple prediction (DIANAmT, miRanda, miRDB, miRWalk, Targetscan)		BCL2, BCL2L1, BCL2L2, BCL2L11, BCL2L12, BCL2L13, BCL2L14			CASP2, CASP6, CASP7, CASP9, CASP10	BRCC3	Many cyclins and CDKs	

*MDA-MB-231*									
hsa-miR-122-5p MIMAT0000421	Binding to 3' UTR		BCL2A1			CASP6		CCNG1, CCNYL1	
	Multiple prediction (DIANAmT, miRanda, miRDB, miRWalk, Targetscan)		Many BCL2L proteins			CASP2, CASP6, CASP10	BRCA1, BRCC3	Many cyclins and CDKs	
hsa-miR-542-3p MIMAT0003389	Binding to 3' UTR							CDKN1A	BIRC5
	Multiple prediction (DIANAmT, miRanda, miRDB, miRWalk, Targetscan)		BCL2, BCL2L15			CASP2, CASP6, CASP10		Many cyclins and CDKs	
hsa-miR-200c-3p MIMAT0000617	Binding to 3' UTR					CASP2		CCNYL1, CCNJ, CDK2	
	Multiple prediction (DIANAmT, miRanda, miRDB, miRWalk, Targetscan)		BCL2, BCL2L11			CASP2, CASP3, CASP10	BRCA1, BRCA2	Many cyclins and CDKs	

Summary table								
MiRNA	Fold regulation		p53	BCL2	FAS	Caspases	BRCA	Cyclins
	MDA-MB-231	MCF-7						
hsa-miR-122-5p	✓ 37.6175		✓	✓		✓	✓	✓
hsa-miR-200c-3p	✓ −8.3784			✓	✓	✓	✓	✓
hsa-miR-542-3p	✓ −11.0553	✓ −8.0937		✓		✓		✓
hsa-miR-125b-5p		✓ −5.7231	✓	✓		✓	✓	✓
hsa-miR-409-3p		✓ 1.6057		✓	✓	✓		✓

## Abbreviations

CDK: cyclin-dependent kinase
miRNA: microRNA
XIAP: X-linked inhibitor of apoptosis protein
IPA: ingenuity pathway analysis
IAP: inhibitor of apoptosis protein
