# Supplementary figures and images for: Role of apoptosis-related miRNAs in resveratrol-induced breast cancer cell death
Source: Cell Death Dis. 2016 Feb 18;7(2):e2104–. doi: 10.1038/cddis.2016.6 (PMC5399194; doi:10.1038/cddis.2016.6)

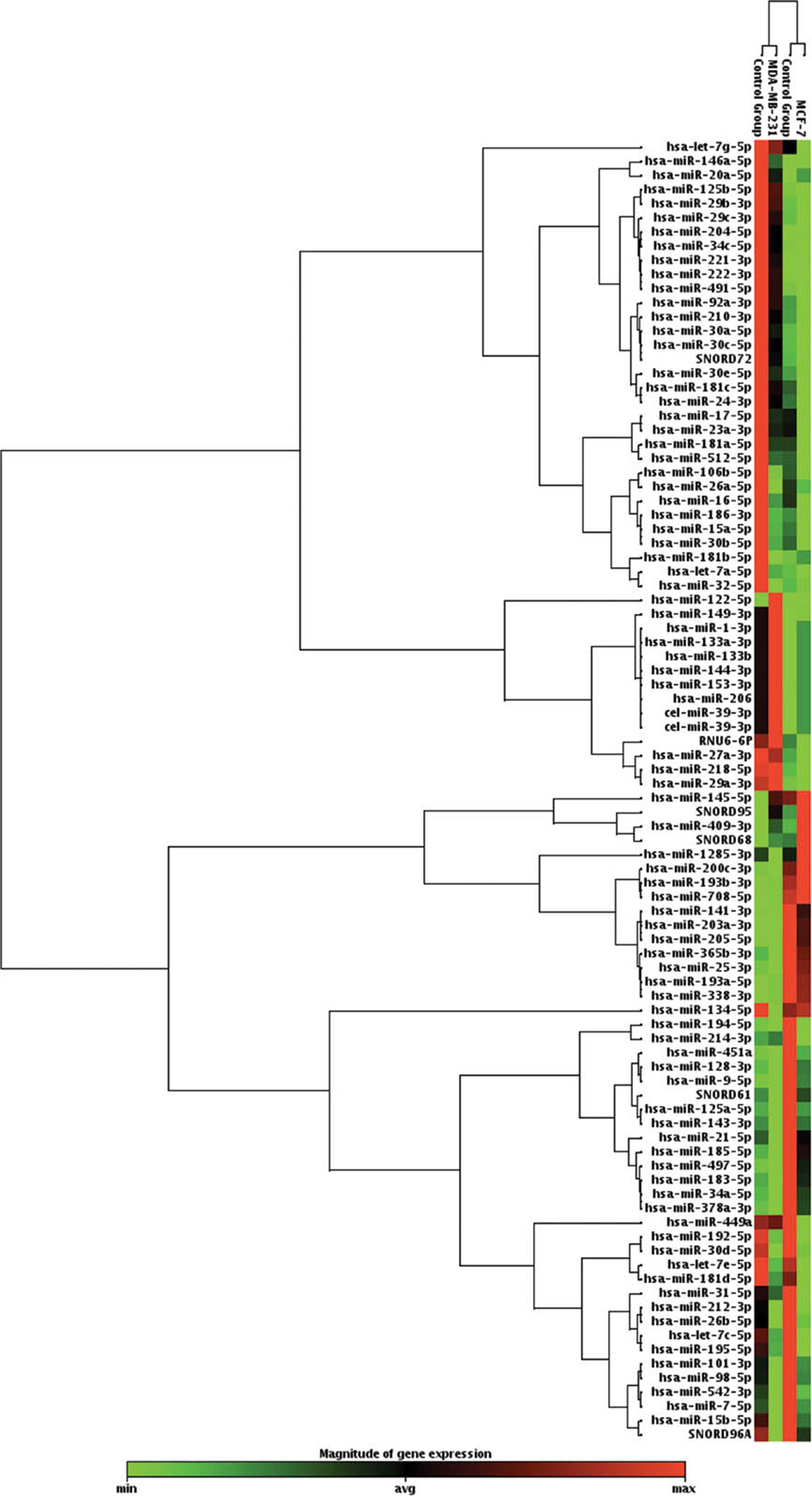

Supplement: Supplementary Figure 1 [file cddis20166x1.tif]
