# Supplementary material for: Role of apoptosis-related miRNAs in resveratrol-induced breast cancer cell death
Source: Cell Death Dis. 2016 Feb 18;7(2):e2104–. doi: 10.1038/cddis.2016.6 (PMC5399194; doi:10.1038/cddis.2016.6)
Supplement: Supplementary Table 1 [file cddis20166x2.docx]

**Supplementary Table 1**

**MCF-7**

| **Position** | **Mature ID** | **Fold Regulation** |
| --- | --- | --- |
| A01 | hsa-let-7a-5p | -1.8879 |
| A02 | hsa-let-7c | -1.4308 |
| A03 | hsa-let-7e-5p | -1.5335 |
| A04 | hsa-let-7g-5p | -2.4911 |
| A05 | hsa-miR-1 | 1.1354 |
| A06 | hsa-miR-101-3p | -2.8615 |
| A07 | hsa-miR-106b-5p | -1.6435 |
| A08 | hsa-miR-122-5p | -1.0843 |
| A09 | hsa-miR-125a-5p | -1.6435 |
| A10 | hsa-miR-125b-5p | -5.7231 |
| A11 | hsa-miR-128 | -2.8615 |
| A12 | hsa-miR-1285-3p | 1.1354 |
| B01 | hsa-miR-133a | 1.1354 |
| B02 | hsa-miR-133b | 1.1354 |
| B03 | hsa-miR-134 | 1.0594 |
| B04 | hsa-miR-141-3p | -1.6435 |
| B05 | hsa-miR-143-3p | -3.0669 |
| B06 | hsa-miR-144-3p | 1.1354 |
| B07 | hsa-miR-145-5p | 1.1354 |
| B08 | hsa-miR-146a-5p | 1.1354 |
| B09 | hsa-miR-149-3p | -1.0117 |
| B10 | hsa-miR-153 | 1.1354 |
| B11 | hsa-miR-15a-5p | -1.6435 |
| B12 | hsa-miR-15b-5p | -1.2456 |
| C01 | hsa-miR-16-5p | -1.8879 |
| C02 | hsa-miR-17-5p | -2.4911 |
| C03 | hsa-miR-181a-5p | -2.3243 |
| C04 | hsa-miR-181b-5p | 1.2169 |
| C05 | hsa-miR-181c-5p | -3.523 |
| C06 | hsa-miR-181d | -2.6699 |
| C07 | hsa-miR-183-5p | -2.1686 |
| C08 | hsa-miR-185-5p | -1.6435 |
| C09 | hsa-miR-186-3p | -2.1686 |
| C10 | hsa-miR-192-5p | -2.8615 |
| C11 | hsa-miR-193a-5p | -1.2456 |
| C12 | hsa-miR-193b-3p | 1.1354 |
| D01 | hsa-miR-194-5p | -3.7758 |
| D02 | hsa-miR-195-5p | -3.2871 |
| D03 | hsa-miR-200c-3p | 1.3042 |
| D04 | hsa-miR-203a | -1.5335 |
| D05 | hsa-miR-204-5p | 1.1354 |
| D06 | hsa-miR-205-5p | -1.5335 |
| D07 | hsa-miR-206 | 1.1354 |
| D08 | hsa-miR-20a-5p | 1.2169 |
| D09 | hsa-miR-21-5p | -1.5335 |
| D10 | hsa-miR-210 | -2.8615 |
| D11 | hsa-miR-212-3p | -3.7758 |
| D12 | hsa-miR-214-3p | -4.0468 |
| E01 | hsa-miR-218-5p | -1.8879 |
| E02 | hsa-miR-221-3p | -1.7615 |
| E03 | hsa-miR-222-3p | -1.5335 |
| E04 | hsa-miR-23a-3p | -2.8615 |
| E05 | hsa-miR-24-3p | -1.8879 |
| E06 | hsa-miR-25-3p | -1.1621 |
| E07 | hsa-miR-26a-5p | -1.2456 |
| E08 | hsa-miR-26b-5p | -3.0669 |
| E09 | hsa-miR-27a-3p | -1.4308 |
| E10 | hsa-miR-29a-3p | -2.0234 |
| E11 | hsa-miR-29b-3p | -2.1686 |
| E12 | hsa-miR-29c-3p | -3.0669 |
| F01 | hsa-miR-30a-5p | -1.335 |
| F02 | hsa-miR-30b-5p | -3.0669 |
| F03 | hsa-miR-30c-5p | -2.0234 |
| F04 | hsa-miR-30d-5p | -1.335 |
| F05 | hsa-miR-30e-5p | -1.8879 |
| F06 | hsa-miR-31-5p | -3.523 |
| F07 | hsa-miR-32-5p | -1.335 |
| F08 | hsa-miR-338-3p | -1.2456 |
| F09 | hsa-miR-34a-5p | -2.4911 |
| F10 | hsa-miR-34c-5p | -3.0669 |
| F11 | hsa-miR-365b-3p | -1.335 |
| F12 | hsa-miR-378a-3p | -2.1686 |
| G01 | hsa-miR-409-3p | 1.6057 |
| G02 | hsa-miR-449a | -1.8879 |
| G03 | hsa-miR-451a | -3.7758 |
| G04 | hsa-miR-491-5p | -1.1621 |
| G05 | hsa-miR-497-5p | -2.1686 |
| G06 | hsa-miR-512-5p | -4.6486 |
| G07 | hsa-miR-542-3p | -8.0937 |
| G08 | hsa-miR-7-5p | -3.7758 |
| G09 | hsa-miR-708-5p | 1.0594 |
| G10 | hsa-miR-9-5p | -3.2871 |
| G11 | hsa-miR-92a-3p | -1.6435 |
| G12 | hsa-miR-98-5p | -2.6699 |
| H01 | cel-miR-39-3p | 1.1354 |
| H02 | cel-miR-39-3p | 1.1354 |
| H03 | SNORD61 | -1.7615 |
| H04 | SNORD68 | 2.1187 |
| H05 | SNORD72 | -1.335 |
| H06 | SNORD95 | 2.1187 |
| H07 | SNORD96A | -1.4308 |
| H08 | RNU6-2 | -1.5335 |
| H09 | miRTC | 1.3978 |
| H10 | miRTC | 1.2169 |
| H11 | PPC | -1.335 |
| H12 | PPC | 2.1187 |

**MDA-MB-231**

| **Position** | **Mature ID** | **Fold Regulation** |
| --- | --- | --- |
| A01 | hsa-let-7a-5p | -4.4898 |
| A02 | hsa-let-7c | -1.203 |
| A03 | hsa-let-7e-5p | -1.4811 |
| A04 | hsa-let-7g-5p | -1.203 |
| A05 | hsa-miR-1 | 1.2599 |
| A06 | hsa-miR-101-3p | -2.9622 |
| A07 | hsa-miR-106b-5p | -2.7638 |
| A08 | hsa-miR-122-5p | 37.6175 |
| A09 | hsa-miR-125a-5p | -1.1225 |
| A10 | hsa-miR-125b-5p | -1.4811 |
| A11 | hsa-miR-128 | -1.5874 |
| A12 | hsa-miR-1285-3p | -1.1225 |
| B01 | hsa-miR-133a | 1.2599 |
| B02 | hsa-miR-133b | 1.2599 |
| B03 | hsa-miR-134 | -5.5277 |
| B04 | hsa-miR-141-3p | -3.1748 |
| B05 | hsa-miR-143-3p | -3.1748 |
| B06 | hsa-miR-144-3p | 1.2599 |
| B07 | hsa-miR-145-5p | 1.9097 |
| B08 | hsa-miR-146a-5p | -3.4027 |
| B09 | hsa-miR-149-3p | 1.2599 |
| B10 | hsa-miR-153 | 1.2599 |
| B11 | hsa-miR-15a-5p | -2.5787 |
| B12 | hsa-miR-15b-5p | -1.203 |
| C01 | hsa-miR-16-5p | -2.2449 |
| C02 | hsa-miR-17-5p | -1.8234 |
| C03 | hsa-miR-181a-5p | -1.9543 |
| C04 | hsa-miR-181b-5p | -3.4027 |
| C05 | hsa-miR-181c-5p | -1.7013 |
| C06 | hsa-miR-181d | -2.2449 |
| C07 | hsa-miR-183-5p | -2.5787 |
| C08 | hsa-miR-185-5p | -1.8234 |
| C09 | hsa-miR-186-3p | -3.9086 |
| C10 | hsa-miR-192-5p | -2.4061 |
| C11 | hsa-miR-193a-5p | 1.1755 |
| C12 | hsa-miR-193b-3p | 1.1755 |
| D01 | hsa-miR-194-5p | -1.1225 |
| D02 | hsa-miR-195-5p | -1.8234 |
| D03 | hsa-miR-200c-3p | -8.3784 |
| D04 | hsa-miR-203a | 1.2599 |
| D05 | hsa-miR-204-5p | -1.9543 |
| D06 | hsa-miR-205-5p | -1.3819 |
| D07 | hsa-miR-206 | 1.2599 |
| D08 | hsa-miR-20a-5p | -1.4811 |
| D09 | hsa-miR-21-5p | -1.7013 |
| D10 | hsa-miR-210 | -1.8234 |
| D11 | hsa-miR-212-3p | -2.4061 |
| D12 | hsa-miR-214-3p | 1.1755 |
| E01 | hsa-miR-218-5p | 1.0234 |
| E02 | hsa-miR-221-3p | -1.8234 |
| E03 | hsa-miR-222-3p | -1.7013 |
| E04 | hsa-miR-23a-3p | -1.8234 |
| E05 | hsa-miR-24-3p | -1.5874 |
| E06 | hsa-miR-25-3p | -1.0473 |
| E07 | hsa-miR-26a-5p | -1.8234 |
| E08 | hsa-miR-26b-5p | -2.2449 |
| E09 | hsa-miR-27a-3p | -1.1225 |
| E10 | hsa-miR-29a-3p | 1.0968 |
| E11 | hsa-miR-29b-3p | -1.4811 |
| E12 | hsa-miR-29c-3p | -1.7013 |
| F01 | hsa-miR-30a-5p | -1.7013 |
| F02 | hsa-miR-30b-5p | -3.9086 |
| F03 | hsa-miR-30c-5p | -1.8234 |
| F04 | hsa-miR-30d-5p | -1.2894 |
| F05 | hsa-miR-30e-5p | -1.9543 |
| F06 | hsa-miR-31-5p | -1.3819 |
| F07 | hsa-miR-32-5p | -4.8121 |
| F08 | hsa-miR-338-3p | 1.2599 |
| F09 | hsa-miR-34a-5p | -2.7638 |
| F10 | hsa-miR-34c-5p | -1.9543 |
| F11 | hsa-miR-365b-3p | -1.7013 |
| F12 | hsa-miR-378a-3p | -1.3819 |
| G01 | hsa-miR-409-3p | 1.2599 |
| G02 | hsa-miR-449a | -1.0473 |
| G03 | hsa-miR-451a | -1.203 |
| G04 | hsa-miR-491-5p | -1.4811 |
| G05 | hsa-miR-497-5p | -2.0946 |
| G06 | hsa-miR-512-5p | -2.9622 |
| G07 | hsa-miR-542-3p | -11.0553 |
| G08 | hsa-miR-7-5p | -4.1892 |
| G09 | hsa-miR-708-5p | 1.0968 |
| G10 | hsa-miR-9-5p | -1.2894 |
| G11 | hsa-miR-92a-3p | -1.4811 |
| G12 | hsa-miR-98-5p | -2.7638 |
| H01 | cel-miR-39-3p | 1.2599 |
| H02 | cel-miR-39-3p | 1.2599 |
| H03 | SNORD61 | -1.3819 |
| H04 | SNORD68 | 1.5511 |
| H05 | SNORD72 | -1.5874 |
| H06 | SNORD95 | 2.0467 |
| H07 | SNORD96A | -1.7013 |
| H08 | RNU6-2 | 1.1755 |
| H09 | miRTC | -1.3819 |
| H10 | miRTC | -7.2938 |
| H11 | PPC | 1.6625 |
| H12 | PPC | 1.0234 |
