# Supplementary material for: Role of apoptosis-related miRNAs in resveratrol-induced breast cancer cell death
Source: Cell Death Dis. 2016 Feb 18;7(2):e2104–. doi: 10.1038/cddis.2016.6 (PMC5399194; doi:10.1038/cddis.2016.6)
Supplement: Supplementary Table 2 [file cddis20166x3.docx]

**Supplementary Table 2**

**MCF-7**

| **Position** | **Mature ID** | **Fold Regulation** |
| --- | --- | --- |
| A01 | hsa-let-7a-5p | -1.5874 |
| A02 | hsa-let-7b-5p | -1.5874 |
| A03 | hsa-let-7c | -1.1225 |
| A04 | hsa-let-7d-5p | -1.3819 |
| A05 | hsa-let-7e-5p | 1.1755 |
| A06 | hsa-let-7f-5p | -1.0473 |
| A07 | hsa-let-7g-5p | -1.5874 |
| A08 | hsa-let-7i-5p | -1.3819 |
| A09 | hsa-miR-1 | 1.0234 |
| A10 | hsa-miR-100-5p | 1.0234 |
| A11 | hsa-miR-107 | -1.0473 |
| A12 | hsa-miR-10a-5p | -1.4811 |
| B01 | hsa-miR-10b-5p | -1.3819 |
| B02 | hsa-miR-125b-5p | -2.2449 |
| B03 | hsa-miR-125b-1-3p | -2.9622 |
| B04 | hsa-miR-128 | -2.0946 |
| B05 | hsa-miR-129-5p | -1.0473 |
| B06 | hsa-miR-130a-3p | 1.0234 |
| B07 | hsa-miR-130b-3p | -1.9543 |
| B08 | hsa-miR-132-3p | -1.7013 |
| B09 | hsa-miR-140-5p | -2.4061 |
| B10 | hsa-miR-141-3p | -1.7013 |
| B11 | hsa-miR-145-5p | -1.3819 |
| B12 | hsa-miR-148a-3p | -2.4061 |
| C01 | hsa-miR-152 | -2.5787 |
| C02 | hsa-miR-155-5p | -1.5874 |
| C03 | hsa-miR-15a-5p | -1.7013 |
| C04 | hsa-miR-15b-5p | -1.4811 |
| C05 | hsa-miR-16-5p | -1.5874 |
| C06 | hsa-miR-17-5p | -1.3819 |
| C07 | hsa-miR-181a-5p | -2.2449 |
| C08 | hsa-miR-181b-5p | -1.5874 |
| C09 | hsa-miR-181c-5p | 1.0234 |
| C10 | hsa-miR-181d | -1.3819 |
| C11 | hsa-miR-182-5p | -2.2449 |
| C12 | hsa-miR-186-5p | -1.5874 |
| D01 | hsa-miR-18a-5p | -2.4061 |
| D02 | hsa-miR-193b-3p | -1.1225 |
| D03 | hsa-miR-195-5p | 1.0234 |
| D04 | hsa-miR-199b-3p | -1.203 |
| D05 | hsa-miR-199a-5p | 2.0467 |
| D06 | hsa-miR-19a-3p | 1.0234 |
| D07 | hsa-miR-19b-3p | -1.4811 |
| D08 | hsa-miR-200a-3p | -2.2449 |
| D09 | hsa-miR-200b-3p | -1.203 |
| D10 | hsa-miR-200c-3p | -1.3819 |
| D11 | hsa-miR-202-3p | -1.7013 |
| D12 | hsa-miR-203a | -1.7013 |
| E01 | hsa-miR-204-5p | 1.0234 |
| E02 | hsa-miR-205-5p | -1.203 |
| E03 | hsa-miR-206 | 1.0234 |
| E04 | hsa-miR-20a-5p | -2.2449 |
| E05 | hsa-miR-20b-5p | -1.203 |
| E06 | hsa-miR-21-5p | -1.4811 |
| E07 | hsa-miR-210 | -1.0473 |
| E08 | hsa-miR-212-3p | -1.9543 |
| E09 | hsa-miR-214-3p | -1.4811 |
| E10 | hsa-miR-22-3p | -1.2894 |
| E11 | hsa-miR-222-3p | -1.4811 |
| E12 | hsa-miR-223-3p | -1.5874 |
| F01 | hsa-miR-25-3p | -1.5874 |
| F02 | hsa-miR-26a-5p | -1.5874 |
| F03 | hsa-miR-26b-5p | -1.203 |
| F04 | hsa-miR-27a-3p | -1.8234 |
| F05 | hsa-miR-27b-3p | -2.0946 |
| F06 | hsa-miR-29a-3p | -1.8234 |
| F07 | hsa-miR-29b-3p | -1.4811 |
| F08 | hsa-miR-29c-3p | -2.5787 |
| F09 | hsa-miR-31-5p | -1.4811 |
| F10 | hsa-miR-328 | -2.2449 |
| F11 | hsa-miR-340-5p | -2.5787 |
| F12 | hsa-miR-424-5p | -3.1748 |
| G01 | hsa-miR-429 | -1.7013 |
| G02 | hsa-miR-485-5p | 1.0234 |
| G03 | hsa-miR-489 | -1.9543 |
| G04 | hsa-miR-495-3p | -2.7638 |
| G05 | hsa-miR-497-5p | -1.7013 |
| G06 | hsa-miR-548c-3p | -1.203 |
| G07 | hsa-miR-607 | 1.0234 |
| G08 | hsa-miR-613 | 2.0467 |
| G09 | hsa-miR-7-5p | -1.8234 |
| G10 | hsa-miR-93-5p | -2.9622 |
| G11 | hsa-miR-96-5p | -1.5874 |
| G12 | hsa-miR-98-5p | -1.9543 |
| H01 | cel-miR-39-3p | 1.0234 |
| H02 | cel-miR-39-3p | 1.0234 |
| H03 | SNORD61 | -1.7013 |
| H04 | SNORD68 | 1.2599 |
| H05 | SNORD72 | 1.1755 |
| H06 | SNORD95 | 1.7818 |
| H07 | SNORD96A | -1.2894 |
| H08 | RNU6-2 | -1.203 |
| H09 | miRTC | 1.5511 |
| H10 | miRTC | 1.2599 |
| H11 | PPC | -1.4811 |
| H12 | PPC | 2.5198 |

**MDA-MB-231**

| **Position** | **Mature ID** | **Fold Regulation** |
| --- | --- | --- |
| A01 | hsa-let-7a-5p | -1.7818 |
| A02 | hsa-let-7b-5p | 1.0473 |
| A03 | hsa-let-7c | 1.0473 |
| A04 | hsa-let-7d-5p | -1.5511 |
| A05 | hsa-let-7e-5p | -1.9097 |
| A06 | hsa-let-7f-5p | -2.5198 |
| A07 | hsa-let-7g-5p | -2.5198 |
| A08 | hsa-let-7i-5p | 1.0473 |
| A09 | hsa-miR-1 | -1.0234 |
| A10 | hsa-miR-100-5p | -1.0234 |
| A11 | hsa-miR-107 | -1.1755 |
| A12 | hsa-miR-10a-5p | 1.0473 |
| B01 | hsa-miR-10b-5p | -1.0968 |
| B02 | hsa-miR-125b-5p | 1.2894 |
| B03 | hsa-miR-125b-1-3p | -3.5636 |
| B04 | hsa-miR-128 | -1.3503 |
| B05 | hsa-miR-129-5p | 1.8234 |
| B06 | hsa-miR-130a-3p | -1.1755 |
| B07 | hsa-miR-130b-3p | -1.0968 |
| B08 | hsa-miR-132-3p | 1.2894 |
| B09 | hsa-miR-140-5p | -2.1936 |
| B10 | hsa-miR-141-3p | -1.5511 |
| B11 | hsa-miR-145-5p | 1.0473 |
| B12 | hsa-miR-148a-3p | -1.5511 |
| C01 | hsa-miR-152 | -1.7818 |
| C02 | hsa-miR-155-5p | -1.0234 |
| C03 | hsa-miR-15a-5p | -2.0467 |
| C04 | hsa-miR-15b-5p | 1.0473 |
| C05 | hsa-miR-16-5p | -1.2599 |
| C06 | hsa-miR-17-5p | -1.2599 |
| C07 | hsa-miR-181a-5p | -1.0234 |
| C08 | hsa-miR-181b-5p | -1.4473 |
| C09 | hsa-miR-181c-5p | -1.4473 |
| C10 | hsa-miR-181d | 1.0473 |
| C11 | hsa-miR-182-5p | 1.3819 |
| C12 | hsa-miR-186-5p | -1.3503 |
| D01 | hsa-miR-18a-5p | -1.3503 |
| D02 | hsa-miR-193b-3p | -1.0234 |
| D03 | hsa-miR-195-5p | -1.0968 |
| D04 | hsa-miR-199b-3p | 6.8053 |
| D05 | hsa-miR-199a-5p | 2.9622 |
| D06 | hsa-miR-19a-3p | 1.0473 |
| D07 | hsa-miR-19b-3p | -1.5511 |
| D08 | hsa-miR-200a-3p | -1.0234 |
| D09 | hsa-miR-200b-3p | -1.3503 |
| D10 | hsa-miR-200c-3p | -1.2599 |
| D11 | hsa-miR-202-3p | -1.1755 |
| D12 | hsa-miR-203a | -1.0234 |
| E01 | hsa-miR-204-5p | -1.6625 |
| E02 | hsa-miR-205-5p | -1.6625 |
| E03 | hsa-miR-206 | -1.0234 |
| E04 | hsa-miR-20a-5p | -13.2998 |
| E05 | hsa-miR-20b-5p | -1.2599 |
| E06 | hsa-miR-21-5p | -1.7818 |
| E07 | hsa-miR-210 | -1.0968 |
| E08 | hsa-miR-212-3p | -1.0234 |
| E09 | hsa-miR-214-3p | 1.5874 |
| E10 | hsa-miR-22-3p | -1.7818 |
| E11 | hsa-miR-222-3p | -1.2599 |
| E12 | hsa-miR-223-3p | 1.3819 |
| F01 | hsa-miR-25-3p | -1.2599 |
| F02 | hsa-miR-26a-5p | -1.6625 |
| F03 | hsa-miR-26b-5p | -1.0234 |
| F04 | hsa-miR-27a-3p | -1.2599 |
| F05 | hsa-miR-27b-3p | -1.7818 |
| F06 | hsa-miR-29a-3p | -1.4473 |
| F07 | hsa-miR-29b-3p | -1.1755 |
| F08 | hsa-miR-29c-3p | -1.1755 |
| F09 | hsa-miR-31-5p | -1.4473 |
| F10 | hsa-miR-328 | 1.3819 |
| F11 | hsa-miR-340-5p | -1.3503 |
| F12 | hsa-miR-424-5p | -1.1755 |
| G01 | hsa-miR-429 | -1.9097 |
| G02 | hsa-miR-485-5p | -1.6625 |
| G03 | hsa-miR-489 | 1.203 |
| G04 | hsa-miR-495-3p | 1.5874 |
| G05 | hsa-miR-497-5p | -1.2599 |
| G06 | hsa-miR-548c-3p | -1.0234 |
| G07 | hsa-miR-607 | -1.0234 |
| G08 | hsa-miR-613 | -1.0234 |
| G09 | hsa-miR-7-5p | -2.3511 |
| G10 | hsa-miR-93-5p | -1.0234 |
| G11 | hsa-miR-96-5p | -2.5198 |
| G12 | hsa-miR-98-5p | -2.0467 |
| H01 | cel-miR-39-3p | -1.0234 |
| H02 | cel-miR-39-3p | -1.0234 |
| H03 | SNORD61 | -32.748 |
| H04 | SNORD68 | 1.3819 |
| H05 | SNORD72 | -2.3511 |
| H06 | SNORD95 | 1.8234 |
| H07 | SNORD96A | -1.0234 |
| H08 | RNU6-2 | -1.3503 |
| H09 | miRTC | -1.7818 |
| H10 | miRTC | 1.3819 |
| H11 | PPC | 1.203 |
| H12 | PPC | -1.4473 |
