# Supplementary material for: Role of apoptosis-related miRNAs in resveratrol-induced breast cancer cell death
Source: Cell Death Dis. 2016 Feb 18;7(2):e2104–. doi: 10.1038/cddis.2016.6 (PMC5399194; doi:10.1038/cddis.2016.6)
Supplement: Supplementary Table 3 [file cddis20166x4.docx]

**Supplementary Table 3**

| **MCF-7** | |
| --- | --- |
| **ID** | **Fold Regulation** |
| hsa-miR-613 | 2.0467 |
| hsa-miR-199a-5p | 2.0467 |
| hsa-miR-125b-5p | -2.2449 |
| hsa-miR-125b-1-3p | -2.9622 |
| hsa-miR-128 | -2.0946 |
| hsa-miR-140-5p | -2.4061 |
| hsa-miR-148a-3p | -2.4061 |
| hsa-miR-152 | -2.5787 |
| hsa-miR-181a-5p | -2.2449 |
| hsa-miR-182-5p | -2.2449 |
| hsa-miR-18a-5p | -2.4061 |
| hsa-miR-200a-3p | -2.2449 |
| hsa-miR-20a-5p | -2.2449 |
| hsa-miR-27b-3p | -2.0946 |
| hsa-miR-29c-3p | -2.5787 |
| hsa-miR-328 | -2.2449 |
| hsa-miR-340-5p | -2.5787 |
| hsa-miR-424-5p | -3.1748 |
| hsa-miR-495-3p | -2.7638 |
| hsa-miR-93-5p | -2.9622 |

| **MDA-MB-231** | |
| --- | --- |
| **ID** | **Fold Regulation** |
| hsa-miR-199b-3p | 6.8053 |
| hsa-miR-199a-5p | 2.9622 |
| hsa-let-7f-5p | -2.5198 |
| hsa-let-7g-5p | -2.5198 |
| hsa-miR-125b-1-3p | -3.5636 |
| hsa-miR-140-5p | -2.1936 |
| hsa-miR-15a-5p | -2.0467 |
| hsa-miR-20a-5p | -13.2998 |
| hsa-miR-7-5p | -2.3511 |
| hsa-miR-96-5p | -2.5198 |
| hsa-miR-98-5p | -2.0467 |

|  |  | | |  | |  |  |
| --- | --- | --- | --- | --- | --- | --- | --- |
| **Common miRNAs** | | | | | |  |  |
| **miRNA** | | | **MCF-7** | **MDA-MB-231** | |  |  |
| hsa-miR-199a-5p | | | 2.0467 | 2.9622 | |  |  |
| hsa-miR-125b-1-3p | | | -2.9622 | -3.5636 | |  |  |
| hsa-miR-140-5p | | | -2.4061 | -2.1936 | |  |  |
| hsa-miR-20a-5p | | | -2.2449 | -13.2998 | |  |  |
